# Electroacupuncture alleviates polycystic ovary syndrome-like symptoms through improving insulin resistance, mitochondrial dysfunction, and endoplasmic reticulum stress via enhancing autophagy in rats

**DOI:** 10.1186/s10020-020-00198-8

**Published:** 2020-07-22

**Authors:** Yan Peng, Liyuan Guo, Anxin Gu, Beibei Shi, Yukun Ren, Jing Cong, Xinming Yang

**Affiliations:** 1 Disease Prevention Center, The First Affiliated Hospital, Heilongjiang University of Chinese Medicine, Harbin, 150040 People’s Republic of China; 2 Department of Gynecological Oncology, Cancer Hospital of Harbin Medical University, Harbin, 150081 People’s Republic of China; 3 Department of Radiation oncology, Cancer Hospital of Harbin Medical University, Harbin, 150081 People’s Republic of China; 4 Graduate School, Heilongjiang University of Chinese Medicine, Harbin, 150040 People’s Republic of China; 5 Department of Obstetrics and Gynecology, The First Affiliated Hospital, Heilongjiang University of Chinese Medicine, Harbin, 150040 People’s Republic of China

**Keywords:** Electroacupuncture, Polycystic ovary syndrome, Insulin resistance, Autophagy, ER stress, Mitochondrial dysfunction

## Abstract

**Background:**

Electroacupuncture (EA), a treatment derived from traditional Chinese medicine, can effectively improve hyperandrogenism and insulin resistance in patients with polycystic ovary syndrome (PCOS), however, its underlying mechanisms remain obscure. This study aimed to investigate whether EA could mitigate PCOS-like symptoms in rats by regulating autophagy.

**Methods:**

A rat model of PCOS-like symptoms was established by subcutaneous injection with dehydroepiandrosterone (DHEA), and then EA treatment at acupoints (ST29 and SP6) was carried out for 5 weeks. To inhibit autophagy in rats, intraperitoneal injection with 0.5 mg/kg 3-MA (an autophagy inhibitor) was performed at 30 min before each EA treatment.

**Results:**

EA intervention alleviated PCOS-like symptoms in rats, which was partly counteracted by the combination with 3-MA. Moreover, DHEA-exposure-induced deficient autophagy in skeletal muscle was improved by EA treatment. EA-mediated improvements in insulin resistance, mitochondrial dysfunction, and endoplasmic reticulum (ER) stress in PCOS-like rats were counteracted by 3-MA pretreatment. Mechanically, EA attenuated autophagy deficiency-mediated insulin resistance in PCOS-like rats via inactivating mTOR/4E-BP1 signaling pathway.

**Conclusions:**

Taken together, our findings indicate that EA treatment ameliorates insulin resistance, mitochondrial dysfunction, and ER stress through enhancing autophagy in a PCOS-like rat model. Our study provides novel insight into the mechanisms underlying the treatment of EA in PCOS, which offers more theoretic foundation for its clinical application.

## Introduction

Polycystic ovary syndrome (PCOS) also known as Stein-Leventhal syndrome, is a complex endocrine and metabolic disorder in gynecology and mainly characterized by hyperandrogenism and insulin resistance (Stener-Victorin et al. 2016). Its incidence is 5–10% among women of reproductive age, which is higher than other infertilities (Batista et al. 2012; Goodarzi et al. 2011). It has been shown that insulin resistance, an important pathological characteristic of PCOS, has been observed in nearly 85% PCOS patients (Stepto et al. 2013). At present, antiestrogen agents are the first-line drugs for treating infertility in women with PCOS. However, these drugs may frequently lose effectiveness and even cause adverse effects (Legro et al. 2007; Zain et al. 2009).

A great deal of animal and human studies proved that acupuncture treatment could effectively relieve the symptoms of PCOS. For example, electroacupuncture (EA) has been shown to modulate the circulating gonadotropin levels and ovarian adiponectin system in PCOS-like rats (Maliqueo et al. 2015). Johansson et al. found that EA intervention could increase ovulation frequency in patients with PCOS (Johansson et al. 2013). Besides, EA has been verified to improve hyperandrogenism and follicular arrest in PCOS-like animals (Shi et al. 2019). In addition, mounting evidence has proved that insulin resistance can be attenuated by EA treatment in PCOS (Johansson et al. 2010; Stener-Victorin et al. 2012; Stener-Victorin et al. 2016). Notwithstanding all this, the detailed mechanisms of EA underlying the protection against PCOS remain ambiguous.

Autophagy dysfunction has been confirmed to contribute to the pathogenesis of PCOS. A previous study showed that the levels of autophagy-related protein DNA damage regulated autophagy modulator 2 was declined in the granulosa cells of PCOS patients (Dai and Lu 2012). The levels of autophagy-related genes *ATG14*, *Beclin-1*, and *ATG-3* were reduced in the endometrium of PCOS patients as compared with the normal controls, which could be remarkably enhanced by metformin treatment (Sumarac-Dumanovic et al. 2017). Moreover, it has been shown that the promotion of autophagy could improve insulin resistance in nonalcoholic steatohepatitis (Amir and Czaja 2011), suggesting that modulation of autophagy tightly connected to insulin resistance. Additionally, overexpression of key autophagy regulatory protein mechanistic target of rapamycin kinase (mTOR) can lead to insulin resistance during the pathological development of PCOS (Liu et al. 2018; Song et al. 2018). Besides, mitochondrial dysfunction and ER stress have been shown to participate in the pathogenesis of PCOS (Azhary et al. 2020; Zeng et al. 2020). Previous studies suggested that autophagy was closely related to mitochondrial dysfunction (Go et al. 2015) and ER stress (Lee et al. 2015). Currently, it is not clear whether EA can mitigate insulin resistance, mitochondrial dysfunction and ER stress through regulating autophagy in PCOS.

In this study, a PCOS-like rat model was established by dehydroepiandrosterone (DHEA)injection. EA-mediated modulation of autophagy and its involvements in insulin resistance, mitochondrial dysfunction, and ER stress in PCOS-like rats were further investigated. Our findings shed light on the novel protective mechanisms of EA in treating PCOS.

## Methods

### Animal model

Four-week-old female Sprague-Dawley (SD) rats were purchased from Chang Sheng biotechnology co., Ltd. (Liaoning, China) and randomly divided into four experimental groups (*n* = 12 per group): control group, PCOS group, PCOS+EA group, and PCOS+EA + 3-MA group. PCOS-like symptoms in rats were induced by daily subcutaneous injection with DHEA (Meilunbio, Dalian, China) at a dose of 6 mg/100 g body weight for 20 consecutive days. The control rats were subcutaneously injected with equal volume sesame oil daily. Immediately after the first injection with DHEA, EA treatment was performed every other day for 5 weeks. According to a previous study (Manneras et al. 2008), the acupuncture points ST29 and SP6 were electrically stimulated at a frequency of 2 Hz and intensity of 0.8–1.3 mA. The EA duration was 15 min in week 1, 20 min in week 2–3, and 25 min in week 4–5. The rats in PCOS+EA + 3-MA group were received intraperitoneal injection with 0.5 mg/kg 3-MA (Aladdin, Shanghai, China) at 30 min before each EA treatment. All rats were subjected to a 12-h fast at the night before the end of the experiment. For each group, six rats were received intraperitoneal injection of 1 U/kg insulin (Aladdin), while the other six rats were injected with equal volume normal saline. 15 min after the injection, all rats were euthanized and then the blood sample, ovary and skeletal muscle tissues were collected for further experiments.

### HE staining

The ovary tissues were fixed in 4% paraformaldehyde, immersed in alcohol gradient, and then embedded in paraffin, followed by cutting into 5-μm sections. After dewaxing and hydration, the sections were successively stained with hematoxylin for 5 min and eosin for 3 min. Finally, the sections were observed under a microscope (Olympus, Japan) at a magnification of 100 × .

### Elisa

The serum levels of testosterone, follicle stimulating hormone (FSH), luteal hormone (LH), and insulin were determined using the commercial ELISA kits (USCN Life Science, Wuhan, China) according to the manufacturer’s protocol.

### Real-time PCR

Total RNA was extracted from ovary or skeletal muscle tissues using a RNApure total RNA isolation kit (Bio Teke, Beijing, China). The concentration of RNA was detected on an ultraviolet spectrophotometer (Nana Drop 2000, Thermo Scientific, USA). Subsequently, cDNA was synthesized using M-MLV Reverse Transcriptase (Takara, Japan). Real time PCR was carried out using Taq HS Perfect Mix (Takara) and SYBR Green (Bio Teke) on an Exicycler™ 96 Real-Time Quantitative Thermal Block ^(^BIONEER, Korea). The primer sequences are presented in Table 1. The mRNA expression level was calculated by 2^-△△CT^ method.

**Table 1:** Oligonucleotide primer sets for real-time PCR

Name	Sequence (5′¬3′)	Length
CYP17 F	TGGAGGTGATAAAGGGTT	18
CYP17 R	CGTCAGGCTGGAGATAGA	18
CYP19 F	GCCTGTCGTGGACTTGGT	18
CYP19 R	TAAATTCATTGGGCTTGG	18
PPARγ F	TACCACGGTTGATTTCTC	18
PPARγ R	AATAATAAGGCGGGGACG	18
PGC-1α F	GAACAAGACTATTGAGCGAACC	22
PGC-1α R	GAGTGGCTGCCTTGGGTA	18
β-actin F	CCACTGCCGCATCCTCTT	18
β-actin R	GGTCTTTACGGATGTCAACG	20

### Western blotting

Total protein was extracted from ovary and skeletal muscle tissues using RIPA buffer (Beyotime, china) supplemented with 1% PMSF (Beyotime). Protein quantification was performed using an Enhanced BCA Protein Assay Kit (Beyotime). Sodium dodecyl sulfate polyacrylamide gel electrophoresis was carried out for protein separation. Then the protein samples were transferred onto PVDF membrane (Thermo Scientific) and blocked in 5% BSA (Biosharp, China). Subsequently, the membranes were incubated with the primary antibodies against CYP17 (1:500, Proteintech, China), CYP19 (1:500, Proteintech), LC3I/II (1:1000, Cell Signaling Technology, USA), Beclin-1 (1:1000, Proteintech), p62 (1:2000, Proteintech), p- mTOR (1:1000, Cell Signaling Technology), mTOR (1:1000, Cell Signaling Technology), 4E-BP1 (1:1000, Cell Signaling Technology), p-4E-BP1 (1:1000, Cell Signaling Technology), PGC-1α (1:1000, Affinity, China), PPARγ (1:1000, Proteintech), GLUT4 (1:500, Proteintech), p-AKT (1:2000, Cell Signaling Technology), AKT (1:1000, Cell Signaling Technology), p-ERK (1:2000, Cell Signaling Technology), ERK (1:1000, Cell Signaling Technology), cytochrome C (1:1000, Abclonal, China), GRP78 (1:1000, Abclonal), ATF4 (1:1000, Abclonal), CHOP (1:1000, Abclonal, China) GRP78 (1:1000, Abclonal), and β-actin (1:2000, Proteintech) at 4 °C overnight. Then the membranes were incubated with Goat anti-rabbit or mouse IgG (1:10000, Proteintech) at 37 °C for 40 min. ECL reagent (7 sea biotech, China) was used to visualize the protein bands.

### Isolation of plasma membrane from skeletal muscle for testing GLUT4 translocation

The plasma membrane of the skeletal muscle tissues was isolated as previously described (Wang et al. 2018). Briefly, the skeletal muscle tissues were rinsed in Solution A containing 250 mmol/L Sucrose, 50 mmol/L Tris, 0.2 mmol/L EDTA, pH 7.4, followed by centrifugation at 120 g for 15 min at 4 °C. Then the mixed supernatant was collected and centrifuged at 9000 g for 20 min at 4 °C. Subsequently, the supernatant was collected and centrifuged at 190,000 g for 60 min at 4 °C. The resulting pellets were re-suspended in 25% sucrose solution and centrifuged at 150,000 g for 16 h at 4 °C. The 25% sucrose layer was collected and centrifuged at 190,000 g for 1 h at 4 °C. The resulting pellets were re-suspended with 100 μL of Solution A. Finally, the samples were subjected to Western blotting for testing GLUT4 translocation.

### Detection of mitochondrial complex enzymes

The activities of mitochondrial complex I and III in the ovarian tissues of rats were assessed using the commercial mitochondrial respiratory chain complex I and III assay kits (Solarbio) following the manufacturer’s instructions.

### Immunofluorescence staining

To assess LC3 expression in skeletal muscle tissues, immunofluorescence staining was carried out. Briefly, the paraffin-embedded skeletal muscle tissues were successively heated in an oven at 60 °C for 30 min, immersed in xylene for 15 min, and hydrated in gradient ethanol. After antigen retrieval and blocking in goat serum (Solarbio, China), the sections were incubated with primary antibody LC3 (1:100, Proteintech) at 4 °C overnight, followed by incubation with Cy3-labeled Goat Anti-Rabbit IgG (1:200, Beyotime) for 60 min. After nuclear staining with DAPI, the sections were observed under an inverted fluorescence microscope (Olympus, Japan).

### Statistical analysis

All experimental data expressed as mean ± standard deviation were statistically analyzed using one-way analysis of variance followed by Tukey post-tests. Significance was recognized as a *P* value less than 0.05.

## Results

### Inhibition of autophagy reversed the beneficial effect of EA on PCOS-like rats

**Fig. 1:**
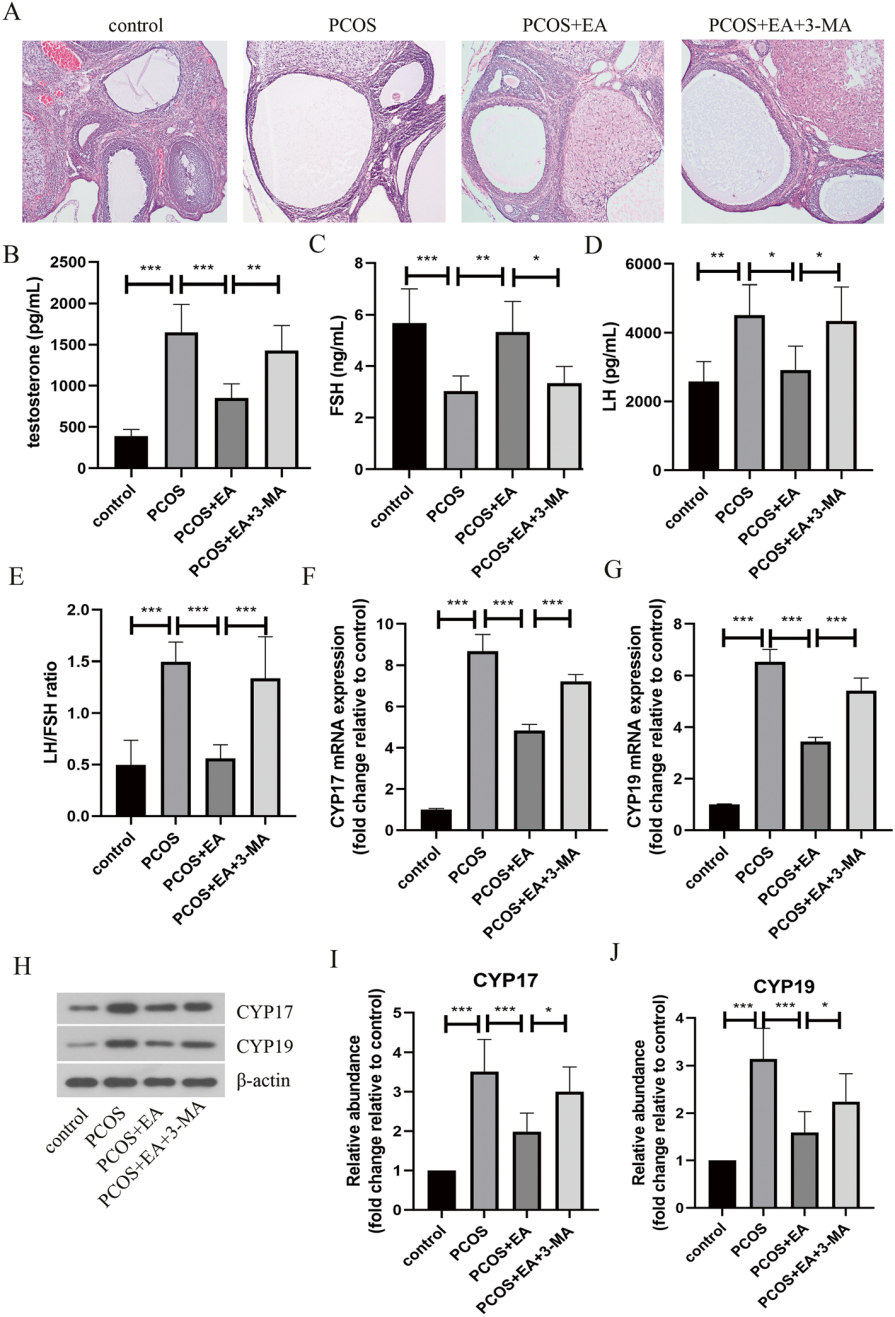
Autophagy inhibition repressed the beneficial effect of EA on PCOS-like rats. **a** The pathological changes of ovarian tissues were determined by HE staining (100×). The serum levels of testosterone (**b**), FSH (**c**), and LH (**d**) were assessed by commercial ELISA kits. (**e**) The ratio of LH/FSH was calculated and shown. The mRNA expression of CYP17 (**f**) and CYP19 (**g**) was detected by real-time PCR. (**h**-**j**) The protein levels of CYP17 and CYP19 were evaluated by Western blotting. The experimental data are presented as mean ± standard deviation (*n* = 6). * *P* < 0.05, ** *P* < 0.01, *** *P* < 0.001, vs the indicated group. EA, electroacupuncture; PCOS, polycystic ovary syndrome; FSH, follicle stimulating hormone; LH, luteal hormone

As shown in Fig. 1a, the pathological manifestations of ovarian tissues after exposure to DHEA were determined by HE staining. The number of follicular cysts of multiple sizes was significantly increased in the ovarian tissues of DHEA-exposed rats, as compared with that of control rats. However, EA treatment could remarkably reduce the number of follicular cysts in PCOS-like rats, which was reversed by autophagy inhibitor 3-MA administration. In addition, the serum levels of testosterone, LH and LH/FSH ratio in PCOS-like rats were remarkably elevated, while the serum FSH level was reduced by almost half (Fig. 1-B-E). Whereas, these changes were attenuated by EA intervention, which were partly restored by 3-MA treatment. Moreover, the increased mRNA levels of CYP17 and CYP19 in the ovarian tissues of PCOS-like rats were downregulated by EA treatment (Fig. 1f&g). As expected, suppression of autophagy by 3-MA reversed EA-mediated changes in CYP17 and CYP19 mRNA levels. These data indicated that EA attenuated PCOS-like symptoms in rats via promoting autophagy.

### Effect of EA on autophagy in PCOS-like rats

**Fig. 2:**
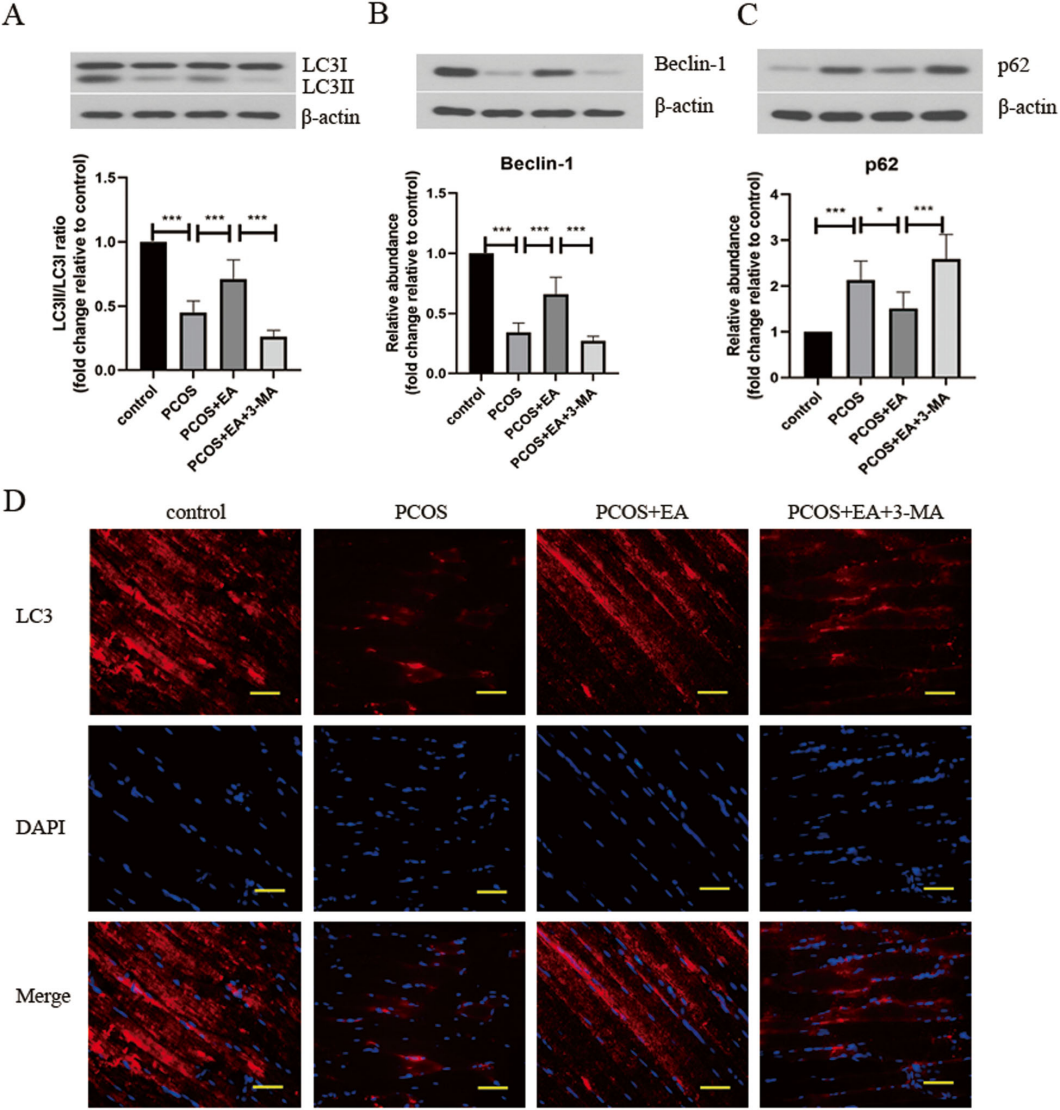
Effect of EA on autophagy in PCOS. The protein levels of LC3I/II (**a**), Beclin-1 (**b**), and p62 (**c**) in the skeletal muscle tissues were detected by Western blotting. **d** The expression of LC3 in skeletal muscle tissues was observed by immunofluorescence staining (400×). Scale bar is 50 μm. The experimental data are presented as mean ± standard deviation (n = 6). * *P* < 0.05, *** *P* < 0.001, vs the indicated group. EA, electroacupuncture; PCOS, polycystic ovary syndrome

Since autophagy promotion participated in the protection of EA against PCOS, the regulation of EA in autophagy was further investigated in PCOS-like rats. As presented in Fig. 2a-c, DHEA exposure-induced upregulation of LC3II/I ratio and Beclin-1 level, but downregulation of p62 level in skeletal muscle tissues were restrained by EA treatment, which were suppressed by 3-MA administration. Moreover, the expression of LC3 in the skeletal muscle tissues was determined by immunofluorescent staining (Fig. 2d). DHEA-challenged rats exhibited lower expression of LC3 in the skeletal muscle tissues compared with control rats, indicating defective autophagy in PCOS-like rats. Treatment with EA significantly restored autophagy by increasing LC3 expression, which was effectively restrained by 3-MA. Therefore, EA treatment promoted autophagy in PCOS-like rats.

### EA restrained insulin resistance in PCOS-like rats via enhancing autophagy

**Fig. 3:**
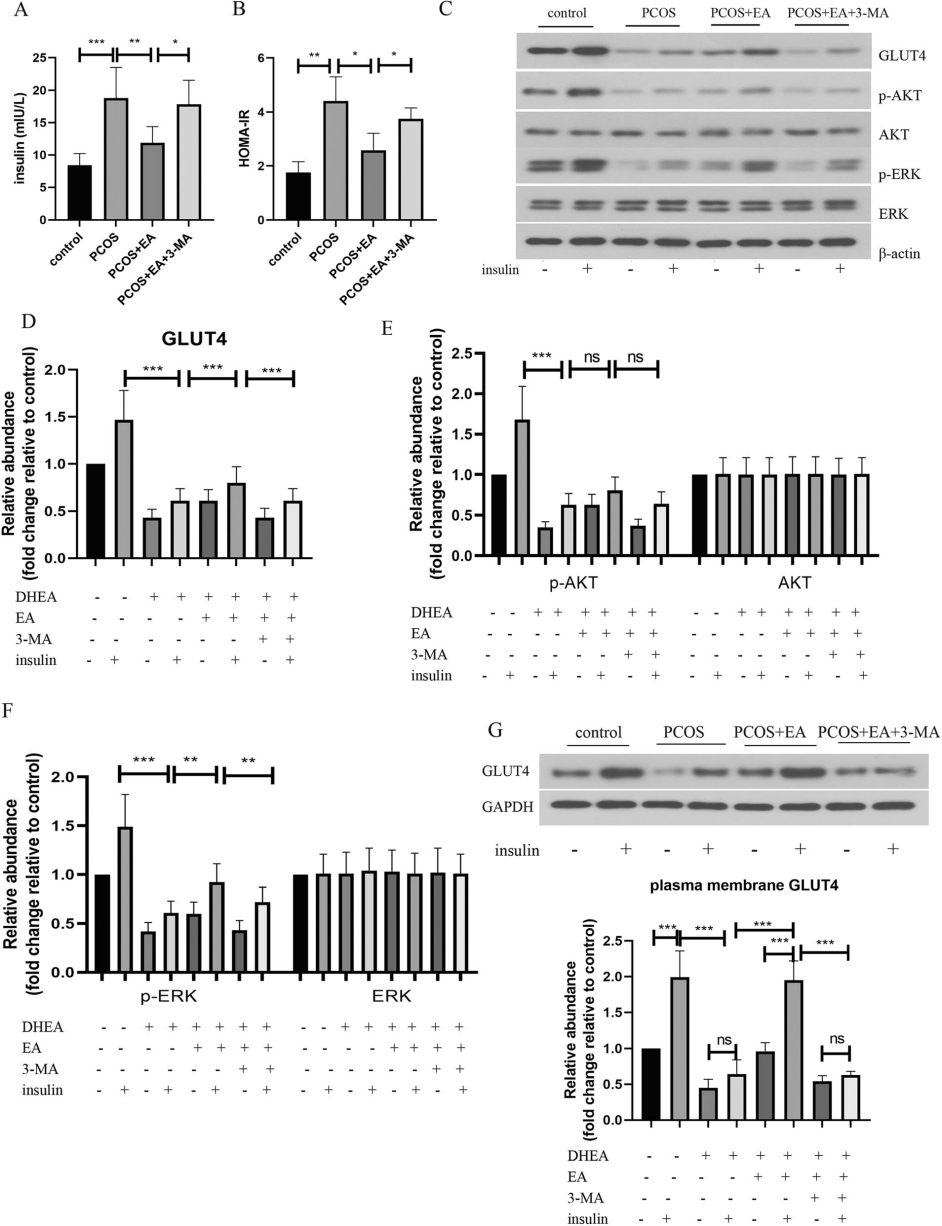
EA restrained insulin resistance in PCOS-like rats via enhancing autophagy. **a** The serum insulin level was detected by a commercial ELISA kit. **b** The HOMA-IR index was detected and shown. **c-f** The protein levels of GLUT4, p-AKT, AKT, p-ERK, and ERK in skeletal muscle tissues were determined by Western blotting. **g** Expression of GLUT4 in plasma membrane of skeletal muscle tissues was detected by Western blotting. The experimental data are presented as mean ± standard deviation (n = 6). * *P* < 0.05, ** *P* < 0.01, *** *P* < 0.001, vs the indicated group. PCOS, polycystic ovary syndrome; HOMA-IR, homeostasis model assessment of insulin resistance

Since insulin resistance is one of important pathological features of PCOS, we further investigated the involvement of autophagy in EA-mediated insulin resistance. As shown in Fig. 3a, the fasting serum insulin level of PCOS-like rats was increased than that in control group, which was effectively downregulated by EA treatment. 3-MA administration repressed EA-mediated downregulation of insulin level. Furthermore, EA intervention led to decreased HOMA-IR index of PCOS-like rats, which was restrained by 3-MA (Fig. 3b). Additionally, insulin sensitivity was evaluated by detecting insulin signaling protein levels in rats treated with or without insulin. As shown in Fig. 3c-f, the protein levels of GLUT4, p-AKT, and p-ERK were obviously decreased in the skeletal muscle tissues of PCOS-like rats. As might be expected, EA treatment could upregulate GLUT4 and p-ERK levels, and 3-MA suppressed these changes. There was no statistical difference in p-AKT level, although p-AKT level appeared to be raised after EA treatment. Insulin-stimulated the translocation of GLUT4 was inhibited by DHEA exposure. However, EA intervention restored GLUT4 translocation in response to insulin, which was reversed by 3-MA administration (Fig. 3g). These results suggested that EA treatment restrained DHEA-induced insulin resistance in skeletal muscle tissues of PCOS-like rats through enhancing autophagy.

### EA attenuated insulin resistance via regulating mTOR/4E-BP1 signaling pathway

**Fig. 4:**
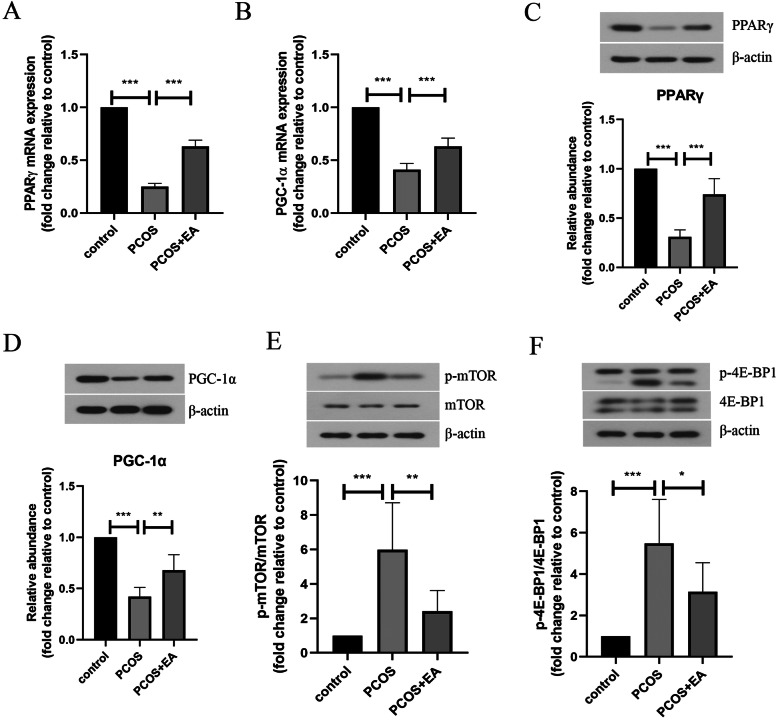
mTOR/4E-BP1 signaling pathway was involved in the protective effect of EA against insulin resistance. The mRNA expression of PGC-1α (**a**) and PPARγ (**b**) in skeletal muscle tissues was assessed by real-time PCR. **c-f** The protein levels of PPARγ, PGC-1α, p-mTOR, mTOR, p-4E-BP1 and 4E-BP1 in skeletal muscle tissues were evaluated by Western blotting. The experimental data are presented as mean ± standard deviation (n = 6). * *P* < 0.05, ** *P* < 0.01, *** *P* < 0.001, vs the indicated group. EA, electroacupuncture; PPARγ, peroxisome proliferator-activated receptor gamma; PGC-1α, PPARγcoactivator-1α. mTOR, mechanistic target of rapamycin; 4E-BP1, 4E-binding protein 1

As presented in Fig. 4a&b, EA treatment remarkably enhanced the mRNA expression of insulin resistance-related factors PGC-1α and PPARγ in PCOS-like rats. Similarly, EA treatment increased the protein levels of PGC-1α and PPARγ in the skeletal muscle tissues of rats exposed to DHEA (Fig. 4C&D). Furthermore, DHEA exposure-induced upregulation of p-mTOR and p-4E-BP1 levels was inhibited by EA treatment (Fig. 4E&F).

### EA improved mitochondrial dysfunction in PCOS-like rats via enhancing autophagy

**Fig. 5:**
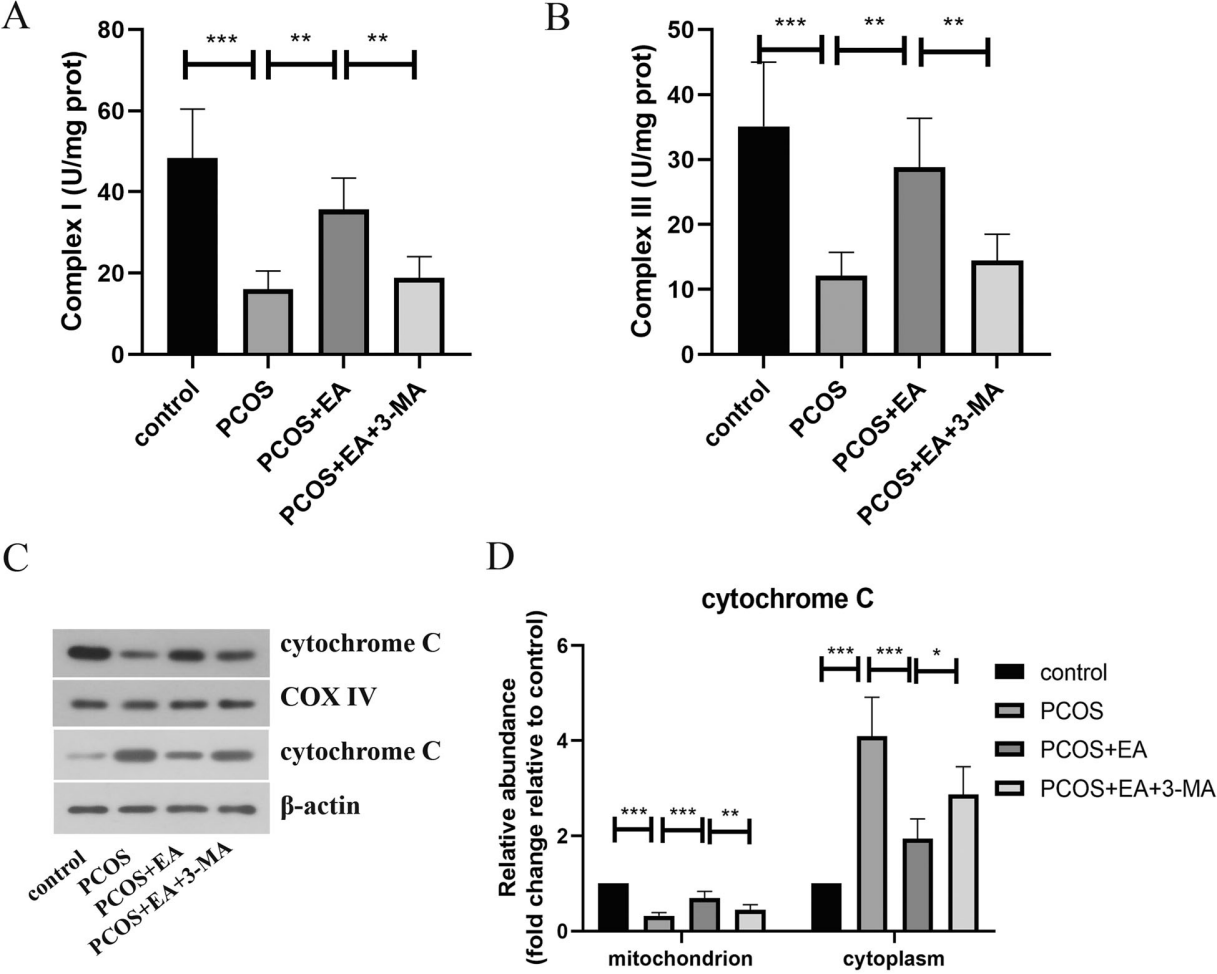
EA improved mitochondrial dysfunction in PCOS rats via enhancing autophagy. The activities of Complex I (**a**) and Complex III (**b**) in ovarian tissues were detected. **c** & **d** Western blotting for determining the protein level of cytochrome C in mitochondrion and cytoplasm of ovarian tissues. The experimental data are presented as mean ± standard deviation (n = 6). * *P* < 0.05, ** *P* < 0.01, *** *P* < 0.001, vs the indicated group. EA, electroacupuncture; PCOS, polycystic ovary syndrome

Next, we investigated the improvement in mitochondrial dysfunction underlying the beneficial effects of EA on PCOS. As presented in Fig. 5A&B, the reduced activities of Complex I and Complex III in the ovarian tissues of PCOS-like rats were evidently improved by EA treatment. Whereas, 3-MA-mediated suppression of autophagy partly reversed the improvement in Complex I and Complex III activities in EA-treated rats. Moreover, Western blotting results showed that EA intervention restrained cytochrome C release from mitochondria into cytoplasm in PCOS-like rats, which was counteracted by 3-MA administration (Fig. 5C&D). Thus, EA improved mitochondrial dysfunction in PCOS-like rats via activating autophagy.

### EA attenuated ER stress in PCOS-like rats via promoting autophagy

**Fig. 6:**
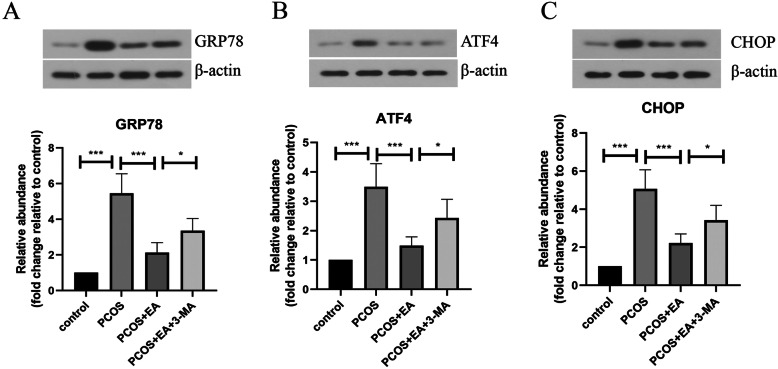
EA attenuated ER stress in PCOS rats via promoting autophagy. **a-c** The protein levels of GRP78, ATF4, and CHOP in ovarian tissues were determined by Western blotting. The experimental data are presented as mean ± standard deviation (n = 6). * *P* < 0.05, *** *P* < 0.001, vs the indicated group. EA, electroacupuncture; PCOS, polycystic ovary syndrome; GRP78, glucose regulated protein 78; ATF4, activating transcription factor 4; CHOP, C/EBP-homologous protein

To further explore whether EA alleviated PCOS-like symptoms in rats via relieving ER stress, the protein levels of ER stress-related proteins GRP78, ATF4, and CHOP were detected by Western blotting. As shown in Fig. 6A-C, a significant increase in the protein levels of GRP78, ATF4, and CHOP in the ovarian tissues of PCOS-like rats was observed, which was declined after receiving EA treatment. Whereas, EA-mediated decrease in GRP78, ATF4, and CHOP levels was significantly repressed by combination with 3-MA. These findings indicated that EA relieved ER stress via promoting autophagy in PCOS-like rats.

## Discussion

PCOS is a complicated disorder of reproductive and endocrine system, which causes serious harm to women all over the world (Moran and Teede 2009). Hyperandrogenism and insulin resistance contribute to the reproductive abnormity in PCOS patients (Rosenfield and Ehrmann 2016). Plenty of clinical trials have suggested that acupuncture may be an effective treatment for PCOS (Cao et al. 2019; Stener-Victorin et al. 2019; Wang et al. 2019). However, the potential mechanisms of acupuncture underlying the treatment of PCOS have not been fully illuminated. In this study, 3-MA-mediated autophagy inhibition counteracted the beneficial effects of EA on PCOS-like symptoms in rats. In addition, our results for the first time demonstrated that the defective autophagy in PCOS-like rats was activated by EA intervention. Moreover, EA ameliorated insulin resistance, mitochondrial dysfunction, and ER stress induced by DHEA via promoting autophagy. Mechanically, EA treatment activated autophagy via regulating mTOR/4E-BP1 signaling pathway.

Previous researches have demonstrated that DHEA-induced PCOS-like animal model displays the clinical characteristics of human PCOS, including hyperandrogenism, aberrant maturation of ovarian follicles and anovulation (Anderson et al. 1992; Lee et al. 1998; Luchetti et al. 2004). A study by Xi et al. showed that DHEA-exposed mice presented whole-body and skeletal muscle insulin resistance, along with autophagy inhibition and mitochondrial injury (Song et al. 2018). Therefore, we selected DHEA to establish a rat model of PCOS-like symptoms, and investigated the role of autophagy in EA-mediated protection against PCOS.

A number of rodent and human studies have proved the effectiveness of EA in treating PCOS. For example, low-frequency EA reversed the epigenetic and transcriptional changes in the adipose tissues of PCOS patients (Kokosar et al. 2018). Electrical or manual acupuncture improved insulin sensitivity by regulating the expression of multiple metabolic Genes and signaling pathways in PCOS model (Benrick et al. 2014). EA enhanced whole-body glucose uptake by activating nervous systems of PCOS women (Benrick et al. 2017). Even so, the detailed mechanisms of EA underlying the protection against PCOS need to be further investigated. Autophagy, as is a physiological process, participates in the pathological processes of multiple diseases, including PCOS. It is reported that the expression of autophagy-related genes was significantly affected in the endometrium of PCOS patients (Sumarac-Dumanovic et al. 2017). Li et al. suggested that autophagy was abnormally activated in ovarian granulosa cells from PCOS patients (Li et al. 2019). Consistently, another research demonstrated the activation of autophagy in PCOS patients and rats (Li et al. 2018). At present, the regulation of autophagy in PCOS is still controversial. In the present study, a decrease in LC3II and Beclin-1 expression, while an increase in p62 expression were found in the skeletal muscle tissues of PCOS-like rats, indicating a suppression of autophagy in PCOS-like rats. Previous studies have reported the regulation of autophagy by acupuncture. For example, EA preconditioning-mediated autophagy promotion could relieve myocardial infarction (Zeng et al. 2018). In addition, in a rat model of Parkinson’s disease, EA treatment could enhance autophagy (Li et al. 2017). In this study, we provided first evidence that EA treatment remarkably enhanced autophagy in the skeletal muscle of PCOS-like rats. Further experiments demonstrated that EA-mediated relief of the hyperandrogenism was restrained by autophagy inhibition. Thus, our data demonstrated that EA alleviated PCOS-like symptoms in rats via activating autophagy.

The close correlation between autophagy and insulin resistance has been confirmed. The deficient autophagy contributes to insulin resistance in obesity (Yang et al. 2010). In addition, the decreased levels of autophagy-related proteins were shown in the liver tissues of mice with insulin resistance (Liu et al. 2009; Yang et al. 2010). Cai et al. showed that autophagy ablation led to restrained insulin action and general insulin resistance in mice (Cai et al. 2018). On the contrary, it was reported that autophagy hyperactivation significantly raised insulin sensitivity in mice treated with high fat diet (Yamamoto et al. 2018). In this study, EA treatment inhibited DHEA-induced insulin resistance as confirmed by decreasing serum insulin level and HOMA-IR index, and this effect was reversed by 3-MA. GLUT-4 is crucial for the transport of glucose to cells in response to insulin. It has been shown that the expression of GLUT-4 was reduced in skeletal muscles of diabetic patients with insulin resistance (Gaster et al. 2001). In addition, insulin-induced AKT activation contributes to the cellular glucose uptake, which is attenuated when insulin resistance occurs (Cai et al. 2018; Yang et al. 2010). It has been documented that the phosphorylation of ERK may be stimulated by insulin independent of AKT pathway (Frendo-Cumbo et al. 2019). In our study, in response to insulin the GLUT4, p-AKT, and p-ERK levels in the skeletal muscle were attenuated in DHEA-challenged group. Although a slight change in these protein levels was observed affected by EA and 3-MA, there were no significant differences among groups. EA intervention restored GLUT4 translocation in response to insulin in PCOS-like rats, which was suppressed by 3-MA administration. More repeated experiments are needed to verify these results. Nevertheless, these findings indicated that EA improved insulin resistance in PCOS-like rats via activating autophagy.

The mTOR kinase has been recognized as a downstream regulator for insulin signaling (Laplante and Sabatini 2012). 4E-BP1, a target of mTOR kinase, can be phosphorylated by mTOR kinase activation. The frequent activation of mTOR has close relation with insulin resistance in patients and animals. Suppression of mTOR activity has been demonstrated to enhance insulin sensitivity (Um et al. 2004). Moreover, the activation of mTOR/4E-BP1 signaling may result in autophagy inhibition in multiple diseases (Follo et al. 2019; Wang and Zhang 2019). Therefore, mTOR/4E-BP1 signaling pathway may participate in the regulation of autophagy-mediated insulin resistance. In line with these observations, our results suggested that EA treatment effectively repressed DHEA-induced hyper-activation of mTOR/4E-BP1 signaling pathway in the skeletal muscle tissues of PCOS-like rats, indicating that EA alleviated insulin resistance in PCOS-like rats via inactivation of mTOR/4E-BP1 signaling pathway.

Recent researches have indicated that mitochondrial dysfunction accelerates the progression of PCOS. For example, Reddy et al. discovered that mitochondria mass was evidently decreased in PCOS (Reddy et al. 2019). In addition, it was verified that mitochondrial dysfunction led to excess production of ROS and accelerated PCOS progression (Ding et al. 2017). More importantly, the mitochondrial copy number was remarkably decreased in PCOS patient with insulin resistance, indicating that mitochondrial dysfunction corelated with PCOS-IR (Ding et al. 2017; Saeed et al. 2019). In the current study, EA treatment effectively attenuated mitochondrial dysfunction in the ovarian tissue of PCOS-like rats. It was revealed that the restoration of mitophagy improved mitochondrial dysfunction and relieved heart failure (Shirakabe et al. 2016). Consistently, our data showed that EA-mediated improvement in mitochondrial dysfunction was partly reversed when autophagy was inhibited. Therefore, our findings suggested that EA attenuated mitochondrial dysfunction via promoting autophagy in the ovarian tissue of PCOS-like rats.

It has been recognized that ER stress is one of pathogenic mechanisms of PCOS (Azhary et al. 2019). ER stress is a biological process that maintains homeostasis, and many molecules and transcription factors are involved in ER stress. The levels of ER stress-related proteins GRP78, ATF4, and CHOP were up-regulated in PCOS patients (Banuls et al. 2017; Takahashi et al. 2017). GRP78 is a marker of ER stress, and the elevated GRP78 level suggests a severe protein misfolding condition. According to our results, an increased expression of GRP78, ATF4, and CHOP in the ovarian tissues of PCOS-like rats was verified. Just as we expected, EA treatment could down-regulate the increased GRP78, ATF4, and CHOP levels in the ovarian tissues of PCOS-like rats. Besides, a close relationship between ER stress and autophagy has been reported by previous studies (Cai et al. 2016; Cybulsky 2017). In this study, our findings demonstrated that inhibition of autophagy by 3-MA partly reversed EA-induced relief of ER stress. These findings revealed that autophagy-mediated ER stress attenuation was involved in the beneficial effect of EA on PCOS-like rats.

We aware of the fact that there are some limitations in the present study. The results indicated that EA and 3-MA treatment had whole body effects, for example altering testosterone, FSH, LH, and insulin levels. Besides, the effects of EA on skeletal muscle and ovary tissues of rats have been studied, but the regulation of autophagy and ER stress in LH/FSH ratio in the brain and insulin resistance in other organs (liver and adipose tissue) has not been investigated in this study. Secondly, a preventing approach of EA was performed in this study. It is better to compare the effect and its related mechanisms of EA between treatment and pretreatment. In our future study, all these issues need to be further explored, which can more comprehensively elucidate the therapeutic mechanisms of EA in treating PCOS.

## Conclusions

Our results demonstrated that EA treatment alleviated PCOS-like symptoms in rats via activating autophagy in skeletal muscle. In addition, activation of autophagy participated in EA-mediated improvement in insulin resistance, mitochondrial dysfunction and ER stress. Mechanistically, EA ameliorated autophagy deficiency-mediated insulin resistance via inactivation of mTOR/4E-BP1 signaling pathway. Our results uncover the novel mechanisms of EA underlying the treatment of PCOS.

## Data Availability

The datasets used or analyzed during the current study are available from the corresponding author on reasonable request.

## Abbreviations

PCOS: Polycystic ovary syndrome
EA: Electroacupuncture
DHEA: Dehydroepiandrosterone
ER: Endoplasmic reticulum
mTOR: Mechanistic target of rapamycin kinase
SD: Sprague-Dawley
FSH: Follicle stimulating hormone
LH: Luteal hormone
HOMA-IR: Homeostasis model assessment of insulin resistance
PPARγ: Peroxisome proliferator-activated receptor gamma
PGC-1α: PPARγcoactivator-1α
4E-BP1: 4E-binding protein 1
GRP78: Glucose regulated protein 78
ATF4: Activating transcription factor 4
CHOP: C/EBP-homologous protein
